# Production of antigen-specific human IgGs by *in vitro* immunization

**DOI:** 10.1186/s12896-016-0253-1

**Published:** 2016-02-24

**Authors:** A. Wijkhuisen, A. Savatier, N. Cordeiro, M. Léonetti

**Affiliations:** University of Paris Diderot, Paris, France; CEA, Institut de Biologie et de Technologie de Saclay (iBiTec-S), Service de Pharmacologie et d’Immunoanalyse (SPI), 91191 Gif sur Yvette, France

**Keywords:** Human antibodies, *In vitro* immunization, Transcriptional transactivator, ZZ domain

## Abstract

**Background:**

We previously developed *in vitro* immunization based on a fusion protein containing the transcriptional transactivator (Tat) of human immunodeficiency virus and a double domain, called ZZ, derived from protein A of *Staphylococcus aureus*. In this approach, naïve human peripheral blood mononuclear cells (PBMCs) trigger a specific IgM antibody (Ab) response in the presence of ZZTat. In the present study, we attempted to raise a specific IgG Ab response.

**Results:**

We found that PBMCs incubated with ZZTat and a mixture containing anti-CD40, IL4 and IL21 secrete anti-Tat IgG Abs in their supernatants, indicating that the cytokine cocktail provides an isotypic switch. Then, we deciphered the Tat determinant involved in the phenomenon and found that it is located in the region 22–57 and that, within this region, the cysteine-rich domain and the basic residues play a crucial role. Finally, we prepared a fusion protein containing a fragment derived from the NY-ESO-1 cancer/testis antigen (Ag) and showed that PBMCs incubated with ZZfNY-ESO-1Tat trigger a specific anti-fNY-ESO-1 IgG Ab response, which demonstrates the possibility of transferring immunizing ability to an Ag unrelated to Tat.

**Conclusion:**

Our ZZTat-based *in vitro* immunization approach that offers the possibility to raise an IgG Ab response against NY-ESO-1 might represent a valuable first stage for the generation of fully human IgG specific Abs.

## Background

Monoclonal antibodies (mAbs) are the fastest growing class of therapeutic agents [1]. Mouse mAbs were first used for therapy in humans [2]. However, their xenogeneic sequences make them prone to elicit immunogenic responses, in particular human anti-mouse antibodies that can reduce their therapeutic efficacy [3]. This drawback led to the development of strategies dedicated to the preparation of mAb-based therapeutics with a high content of human sequences [4, 5]. Chimerization and humanization approaches have addressed this issue with substantial efficiency since, currently, they account for 22 of the 30 mAbs approved for clinical applications [6]. However, some patients still develop anti-Ab responses against these engineered molecules [7]. Therefore, to address this immunogenic drawback more efficiently, numerous studies have been aimed at generating Ab-based molecules containing fully-human sequences [8]. Approaches have been developed using phage display methods [9, 10] or transgenic mice containing human immunoglobulin genes [11]. Other strategies are based on the isolation of human B-lymphocytes from human donors [12, 13]. The latter approaches, which are of proven efficiency with immune donors [12], are more difficult to implement when donors are naïve for the target Ag, due to the difficulty of triggering stimulation of specific B-lymphocytes *in vitro*. Several research groups have developed various *in vitro* immunization procedures for the preparation of specific immortalized human B-lymphocytes using various fusion partners [14, 15] or Epstein-Barr virus [16–18]. However, these techniques have a poor yield and reproducibility [19–21]. Furthermore, the protocols reported are complex since they require several steps for depletion or enrichment of various sub-populations [15, 22].

We recently considered the possibility of improving *in vitro* immunization and making protocols simpler. As various cytokines are usually required to achieve efficient stimulation of naïve lymphocytes by an antigen [23], we decided to assess whether an Ag exhibiting several biological activities related to the triggering of the immune response, in particular activities that are reminiscent of those triggered by cytokines and adjuvants, can be used for *in vitro* immunization. This Ag is the transcriptional transactivator of HIV-1, called Tat101, which is a small protein of 86 to 101 residues [24]. Previous work suggests that Tat can induce chemotaxis of monocytes [25] and secretion of proinflammatory cytokines [26]. Furthermore, Tat was proposed to target monocyte-derived dendritic cells, and to enhance their maturation, function, and Ag-specific T-cell responses [27]. In addition, this protein can reprogram immature dendritic cells to express chemoattractants for activated T cells and macrophages [28]. Furthermore, Tat can bind heparan sulfate proteoglycans, thus increasing the ability of an antigen to stimulate T-helper cells [29]. Lastly, in mice, Tat is able to raise a humoral immune response in the absence of adjuvant [30] and the determinant controlling this unusual property can be used to confer the adjuvant-free characteristic on another Ag [31].

To assess whether Tat is able to trigger a humoral immune response *in vitro* and if we can transfer this ability to other Ags, we used a genetic system that allows the expression of one or more Ags in tandem [32]. In this system, the Ags are fused to a double Ig-binding domain, called ZZ, which is derived from protein A of *Staphylococcus aureus* [33]. In a previous study, we prepared a ZZ-fusion protein, named ZZTat101, containing the transcriptional transactivator of HIV-1 and showed that i) ZZTat101 is able to trigger the production of anti-Tat101 antibodies by PBMCs in the absence of cytokines, indicating that the fusion protein behaves as a surrogate of some of these molecules [34]; ii) the Ab response does not require a previous depletion of immunosuppressive cells and is therefore simple to implement; iii) Tat101 can stimulate the *in vitro* immune response only when covalently coupled to ZZ and when the Tat cysteines are present in the fusion protein, demonstrating that these residues play a crucial role in the phenomenon; iv) the covalent coupling of a hapten to ZZTat101 enables PBMCs to trigger an anti-haptenic Ab response, indicating that the ability to trigger a humoral immune response *in vitro* can be conferred on another Ag. However, during these experiments, we only found specific Abs of the IgM class, indicating that ZZTat is able to initiate the signals required to trigger a primary immune response, but failed to induce the isotypic switch.

In the present study, we addressed several issues. Firstly, can we find an optimized *in vitro* immunization procedure that enables ZZTat101 to trigger a specific anti-Tat IgG Ab response? Secondly, what is the frequency of the specific B-lymphocytes elicited? Thirdly, is it possible to transfer to an unrelated peptide Ag the ability to trigger an *in vitro* IgG Ab response? We assessed this last question using a fusion protein containing ZZ, Tat101 and a tumor non-haptenic peptide Ag.

## Results

### An anti-Tat101 IgG Ab response is triggered *in vitro* when PBMCs are incubated with ZZTat101 and a cytokine cocktail containing IL-21 + IL-4 + anti-CD40

**Fig. 1:**
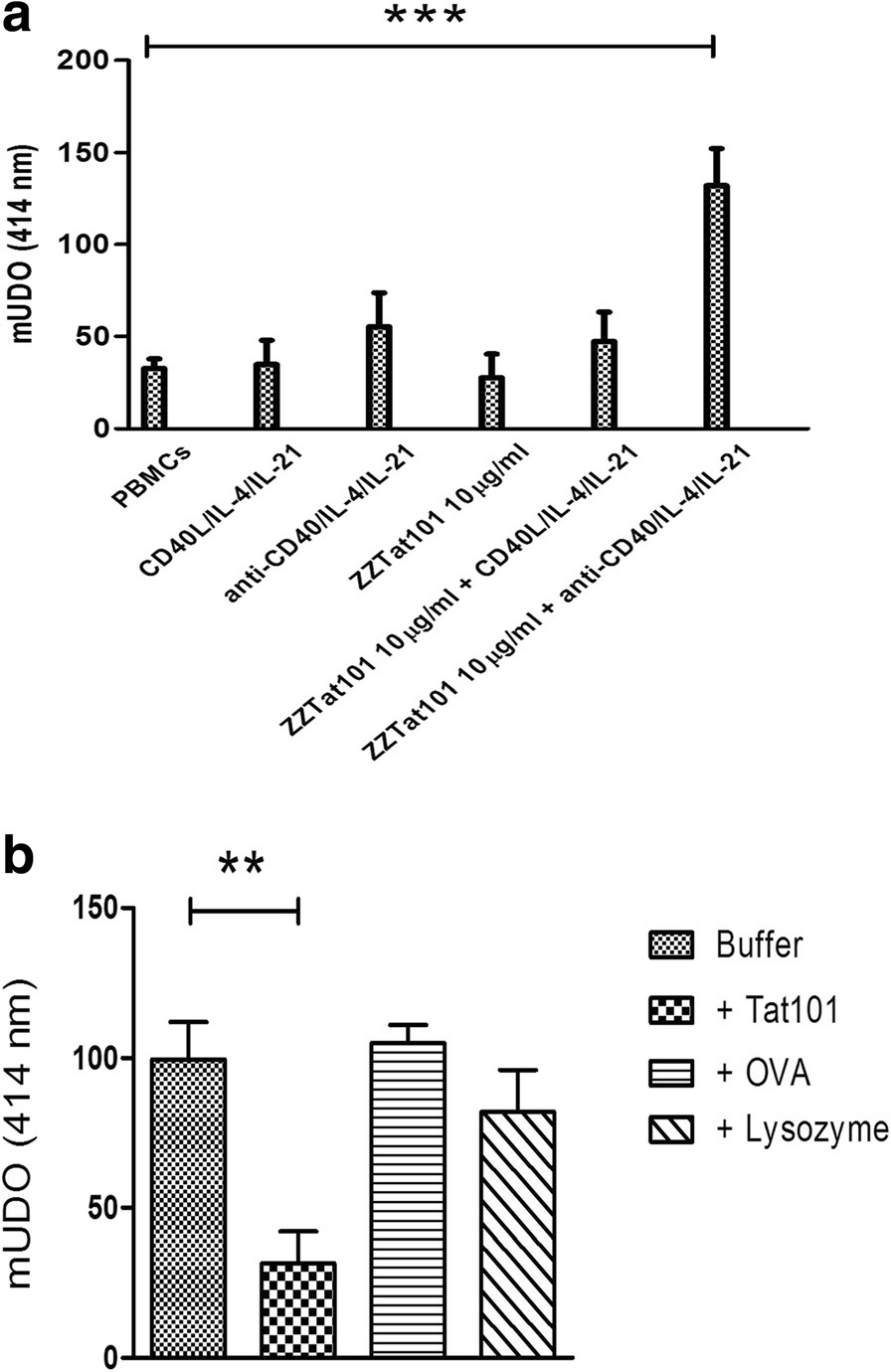
**a** An anti-Tat IgG Ab response is raised *in vitro* when PBMCs are incubated with ZZTat101 and a cytokine/activator cocktail. PBMCs (5 × 10^5^ cells per well) were incubated with or without CD40L/IL-4/IL-21, anti-CD40/IL-4/IL-21, ZZTat101, ZZTat101 + CD40L/IL-4/IL-21, ZZTat101 + anti-CD40/IL-4/IL-21, respectively. Then, supernatants were collected and added to Tat101-coated plates. After overnight incubation, a peroxidase-conjugated anti-human IgG was added and enzymatic activity was determined using ABTS as substrate. **b** The anti-Tat IgG Ab response is specific to Tat101. Supernatants from PBMCs incubated with ZZTat101 + anti-CD40/IL-4/IL-21 were mixed with either a dilution buffer or a fixed amount (10 μg/mL) of three different Ags (Tat101, ovalbumin, lysozyme) and incubated in Tat101-coated plates. Presence of anti-Tat IgG was assessed as in A (**: *p* < 0.01; ***: *p* < 0.001)

As we previously observed that the anti-Tat antibodies secreted by PBMCs after their incubation with ZZTat101 are only of the IgM class [34], we assessed whether addition of cytokines known to promote an isotype switch can favor secretion of anti-Tat IgGs. We selected a mixture containing either IL-21 + IL-4 + CD40L or IL-21 + IL-4 + anti-CD40 since previous reports showed that IL21 together with IL4 regulates an isotype switch by human naive B cells, when cultured with CD40L [35] or anti-CD40 mAb [36]. To investigate the efficiency of this approach, we incubated PBMCs in the presence or absence of ZZTat101 and/or cytokine/activator cocktails. Eleven days later, we collected the supernatants (SNs) and examined the presence of IgG able to bind Tat101-coated plates. As shown in Fig. 1a, binding is poor with SN from PBMCs incubated in the absence of Ag and activator. When we compared this binding with that provided by the other SNs, we did not find a significant difference for those resulting from the incubation of PBMCS with IL-21/IL-4/CD40L, IL-21/IL-4/anti-CD40 and ZZTat101, respectively. Therefore, these results indicated that the cytokine/activator cocktails as well as ZZTat101 are not able to trigger an anti-Tat IgG response when incubated separately. We also found an absence of statistical difference for SN from PBMCs incubated with IL-21/IL-4/CD40L and ZZTat101, indicating that this combination is also inefficient. In contrast, we observed significantly higher binding with SN from PBMCs incubated with IL-21/IL-4/anti-CD40/ZZTat101. Furthermore, binding of this latter SN is significantly lower in plates coated with an antigen unrelated to Tat (not shown), which suggest that the increased interaction to Tat-coated plates comes from specific IgGs. Therefore, altogether these results indicate that an anti-Tat101 isotype switch is caused when PBMCs are incubated with IL-21/IL-4/anti-CD40 and ZZTat101.

To characterize the specificity of the IgG antibodies bound to Tat101-coated plates, we assessed binding in the presence of a fixed amount (10 μg/mL) of three unrelated soluble antigens. We observed that soluble Tat101 causes significant 70 % inhibition of binding, whereas neither ovalbumin (OVA) nor lysozyme significantly inhibits IgG binding to plates (Fig. 1b). Therefore, these results indicate that binding to plates is mainly caused by anti-Tat101-specific IgGs present in the SN of PBMCs incubated with IL-21/IL-4/anti-CD40/ZZTat101.

### Characterization of the molecular determinant controlling the ability to raise the specific humoral response

#### Elicitation of the anti-Tat IgG Ab response requires the covalent coupling of ZZ and Tat101

**Fig. 2:**
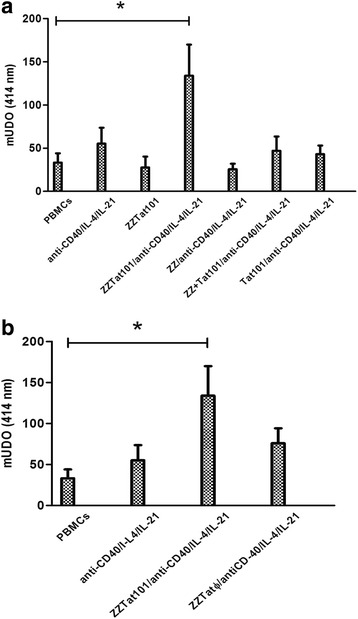
**a** The covalent coupling of Tat101 to ZZ is absolutely required to trigger an anti-Tat IgG Ab response. PBMCs were incubated with or without anti-CD40/IL-4/IL-21, ZZTat101, ZZTat101 + anti-CD40/IL-4/IL-21, ZZ + anti-CD40/IL-4/IL-21, ZZ + Tat101 + anti-CD40/IL-4/IL-21, Tat101 + anti-CD40/IL-4/IL-21, respectively. Then, supernatants were collected and added to Tat101-coated plates. Presence of anti-Tat IgG was assessed as in Fig. 1a. **b** The anti-Tat IgG response requires preservation of the Tat lysines. PBMCs were incubated with or without anti-CD40/IL-4/IL-21, ZZTat101 + anti-CD40/IL-4/IL-21, ZZTat101ϕ + anti-CD40/IL-4/IL-21, respectively. Presence of anti-Tat IgG was assessed in supernatants as in Fig. 1a (*: *p* < 0.05)

To decipher the relative contributions of ZZ and Tat101 to the ability to raise and an IgG Ab response, we incubated PBMCs with the activating cocktail (IL-21/IL-4/anti-CD40) and various antigenic mixtures. Then we compared the ability of the SNs resulting from these incubations to bind Tat101-coated plates. For the SNs from PBMCs respectively incubated with Tat101, ZZ, and ZZ + Tat101, we found a low optical density that did not differ significantly from that of the SNs from PBMCs incubated either without Ag or without Ag and activator (Fig. 2a). In contrast, we observed a significantly higher optical density for the SN resulting from the incubation with ZZTat101/IL-21/IL-4/anti-CD40. Therefore, these results indicate that i) ZZ and Tat101 cannot trigger the IgG Ab response when they are used as free molecules, ii) covalent coupling of Tat101 to ZZ is absolutely required for the phenomenon.

### Elicitation of the anti-Tat IgG Ab response requires the preservation of the basic region of Tat

As Tat101 contains many lysines, which play a role in numerous biological activities of the protein [37], we investigated their role in the *in vitro* immune response using a ZZTat101 fusion protein, called ZZTat101Ø, in which we had previously acetylated the lysine residues using an excess of acetic anhydride. We incubated PBMCs in the presence or absence of either ZZTat101Ø or ZZTat101, and assessed the ability of the SNs to bind Tat101-coated plates. As shown in Fig. 2b, the optical density observed with SNs from incubation with ZZTat101Ø did not differ from that observed with supernatants from PBMCs incubated in the absence of antigen, while it was significantly higher with the SNs resulting from incubation with ZZTat101. Therefore, these results indicate that acetylation of ZZTat101 abrogates the ability to trigger the immune response *in vitro* and that some lysine residues of the fusion protein contribute to the phenomenon.

### The 22–57 region of Tat suffices to raise the specific humoral response and its cysteine-rich region controls the triggering of the IgG response

**Fig. 3:**
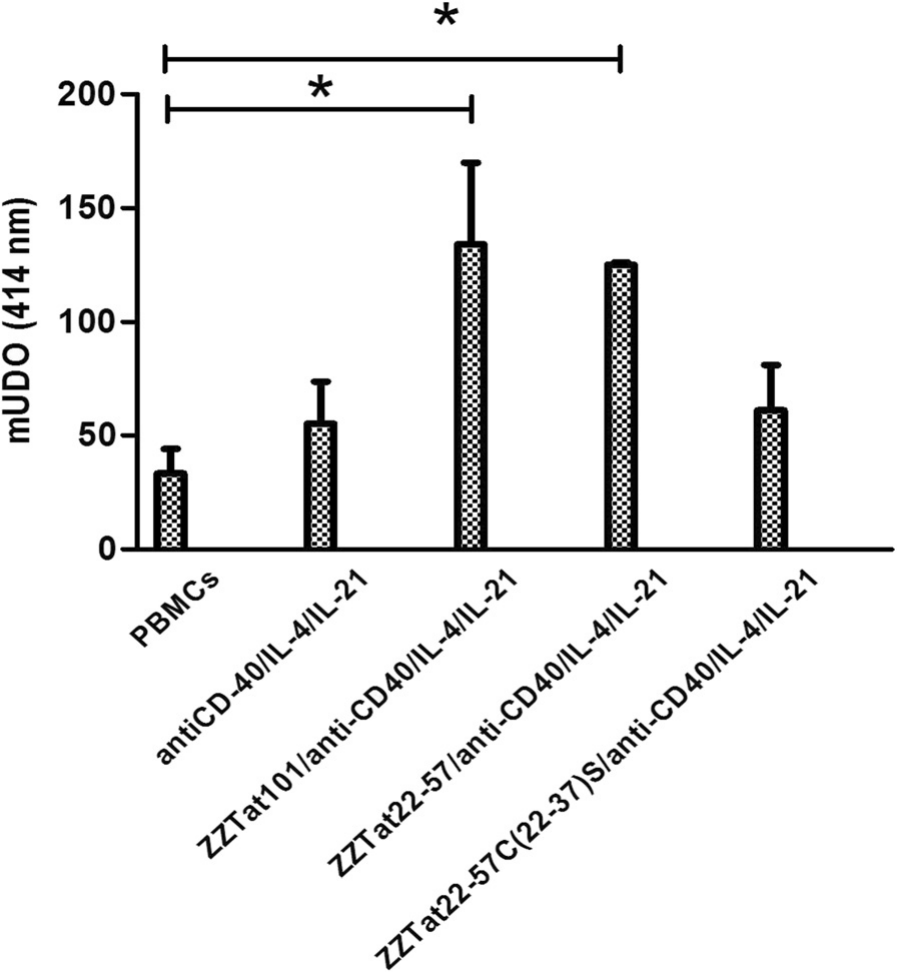
The 22–57 region of Tat suffices to raise an IgG response and preservation of the cysteine residues is absolutely required for the phenomenon. PBMCs were incubated for seven days with or without anti-CD40/IL-4/IL-21, ZZTat101 + anti-CD40/IL-4/IL-21, ZZTat22-57 + anti-CD40/IL-4/IL-21, ZZTat22-57C(22–37)S + anti-CD40/IL-4/IL-21, respectively. Then, supernatants were collected and added to Tat101-coated plates. Presence of anti-Tat IgG was subsequently assessed as described in Fig. 1a

In order to delineate more thoroughly the Tat region involved in the ability to raise a humoral immune response *in vitro*, we prepare two ZZTat mutants. The first, called ZZTat22–57, was selected since i) we showed that a peptide derived from the 22–57 sequence of Tat is able to raise an adjuvant-free antibody response in mice [30], indicating that it can initiate the immune response, at least *in vivo*; ii) we observed that this fusion protein can trigger an anti-Tat IgM response *in vitro* [34]. The second, called ZZTat101_C(22–37)S_, corresponds to a ZZTat22–57 mutant in which the 7 cysteine residues have been replaced by serines. We selected this mutant since i) the Tat22–57 region contains a cysteine-rich region (residues 22 to 37) controlling the capacity to trigger an adjuvant-free immune response in mice [30]; ii) we observed that this fusion protein cannot trigger an anti-Tat IgM response *in vitro* [34]. We incubated ZZTat101, ZZTat22–57 and ZZTat101_C(22–37)S_ in the presence of PBMCs, collected the SNs and assessed their ability to bind to Tat101-coated plates. For the SNs from ZZTat101 and ZZTat22–57 incubations, we found close optical density values that are significantly higher than that of the SNs from PBMCs incubated in the absence of Ag, indicating that the region 22–57 of Tat101 suffices to trigger an *in vitro* immune response (Fig. 3). In contrast, when we compared the SN from ZZTat101_C(22–37)S_ incubation with the SNs from PBMCs incubated in the absence of Ag, we did not find a significant difference of optical density, indicating that the cysteine-rich region of Tat101 controls the triggering of the IgG immune response.

### Determination of the frequency of Tat101-specific IgG-secreting B-lymphocytes

**Fig. 4:**
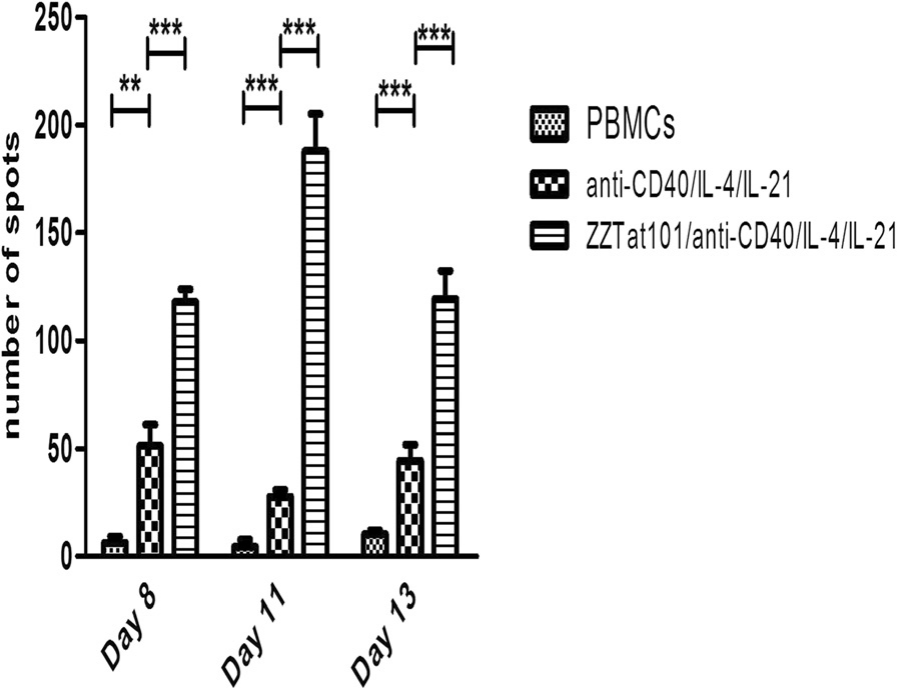
An optimal anti-Tat specific B-lymphocyte response was reached after 11 days of *in vitro* immunization. PBMCs were incubated for eight, eleven or thirteen days in the presence or absence of anti-CD40/IL-4/IL-21, ZZTat101 + anti-CD40/IL-4/IL-21, respectively. Then, PBMCs were transferred from culture plates to ELISPOT plates previously coated with Tat101. After an additional 24-h incubation at 37 °C, a biotinylated anti-human IgG was added. Spot-forming cells were then detected using a streptavidin-alkaline phosphatase conjugate and NBT/BCIP as substrate. Spot-forming cells were quantified with an automated ELISPOT reader

In order to determine the number of Tat101-specific IgG-secreting B-lymphocytes during the *in vitro* immunization experiments, we assessed the number of Tat-specific SFCs in PBMCs incubated for different times with IL-21/IL-4/anti-CD40 in the presence or absence of ZZTat. In these experiments, the last day of incubation we transferred the PBMC samples from culture plates to ELISPOT plates previously coated with Tat101 and measured the number of IgG anti-Tat SFCs after an additional 24-h incubation at 37 °C. As shown in Fig. 4, a number of spots ranging from 25 to 50 was observed in samples from PBMCs incubated only with IL-21/IL-4/anti-CD40. This amount is significantly higher than that found in samples from control PBMCs incubated in the absence of the activating cocktail, indicating that the cocktail induces some B-lymphocytes to secrete IgGs endowed with the ability to bind Tat-coated plates. This behavior could be due to non-specific interactions or to the presence of B-lymphocytes secreting polyreactive IgGs of low affinity that are detected due to the high sensitivity of the ELISPOT as compared to the ELISA methodology. Finally, when we compared PBMCs incubated only with IL-21/IL-4/anti-CD40 with PBMCs incubated with IL-21/IL-4/anti-CD40 and ZZTat101, we found that the latter cells contained a significant 2.5- to 6-fold higher amount of SFCs, indicating that the activating mixture triggers a specific B-lymphocyte response that peaks after 11 days of *in vitro* immunization.

### The ZZ-Tat-based approach can confer on fNY-ESO-1 the ability to trigger an IgG Ab response *in vitro*

**Fig. 5:**
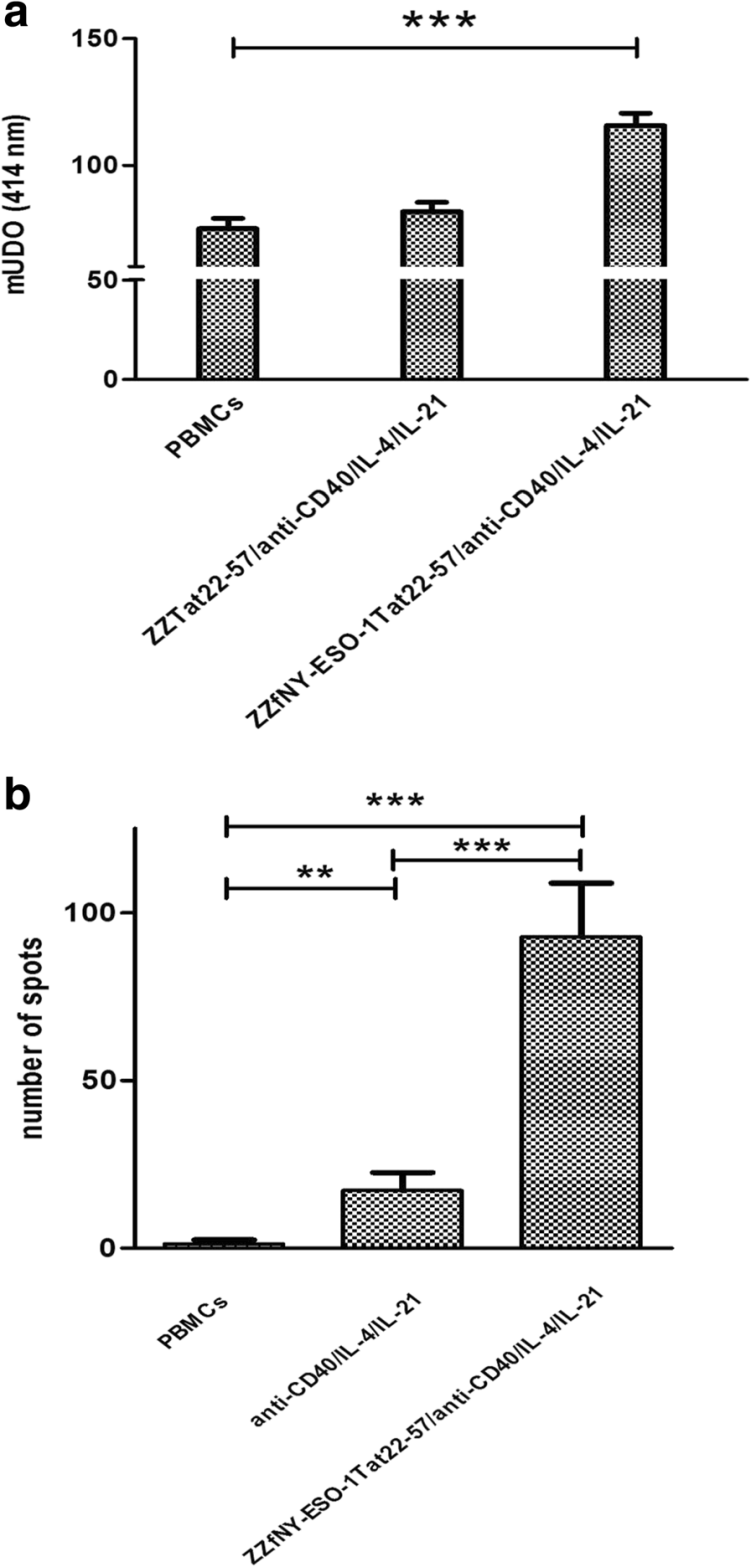
ZZTat101 is able to confer on fNY-ESO-1 the ability to induce B-lymphocytes to secrete anti-fNY-ESO-1 IgG antibodies. **a** PBMCs were incubated for eleven days without anti-CD40/IL-4/IL-21, with ZZTat22-57 + anti-CD40/IL-4/IL-21 or ZZfNY-ESO-1Tat22-57 + anti-CD40/IL-4/IL-21, respectively. Then, supernatants were collected and added to anti-human IgG-coated plates. After an incubation of 2 h, biotinylated f-NY-ESO-1 was added to each well. After overnight incubation, an acetylcholinesterase-labeled streptavidin conjugate was added and enzymatic activity was determined using Ellman's reagent. **b** PBMCs were incubated for eleven days with or without anti-CD40/IL-4/IL-21, ZZfNY-ESO-1Tat22-57 + anti-CD40/IL-4/IL-21, respectively. PBMCs were transferred from culture plates to ELISPOT plates previously coated with fNY-ESO-1. After an additional 24-h incubation at 37 °C, a biotinylated anti-human IgG was added. Spot-forming cells were then detected using a streptavidin-alkaline phosphatase conjugate and NBT/BCIP as substrate. Spot-forming cells were quantified with an automated ELISPOT reader

Since the ZZ-fusion system allows the production of one or more antigens in tandem [32], we decided to build a ZZ-fusion protein containing both Tat22-57 and a human tumor antigen. We selected NY-ESO-1, a protein which is expressed in a wide range of cancers and whose normal tissue expression is generally restricted to testis [38]. However, as a previous report suggested that the whole NY-ESO-1 protein is poorly expressed in *E. coli* [39], we decided to use a NY-ESO-1 fragment of 44 residues that we called fNY-ESO-1. We used peptide chemistry to synthesize the free fragment and molecular biology to produce the fusion protein, called ZZfNY-ESO-1Tat22-57. Then, in *in vitro* immunization experiments we assessed whether the stimulating capacity of ZZTat22-57 can confer on fNY-ESO-1 the ability to induce B-lymphocytes to secrete IgG anti-fNY-ESO-1 antibodies. We incubated PBMCs in the presence or absence of ZZTat22-57 or ZZfNY-ESO-1Tat22-57 and cytokine/activator cocktail. Eleven days later, we collected the SNs in order to examine by enzyme immunoassay the presence of IgGs able to bind fNY-ESO-1-coated plates. As shown in Fig. 5a, binding of SN from PBMCs incubated with ZZfNY-ESO-1Tat22-57 and the cytokine/activator cocktail was significantly higher than binding of SN from PBMCs incubated with ZZTat22-57 and the cytokine/activator cocktails. We also assessed the number of fNY-ESO-1-specific IgG-secreting B-lymphocytes by the ELISPOT technique. We observed a very low number of spots for control PBMCs incubated in the absence of Ag and of the activating cocktail (Fig. 5b). We found a significantly higher number of spots for PBMCs incubated with IL-21/IL-4/anti-CD40. We assume that this corresponds either to non-specific activation or to the presence of B-lymphocytes secreting polyreactive IgGs since we did not detect any anti-fNY-ESO-1 Ab by ELISA (see Fig. 5a). We found a significantly higher number of spots when cells were incubated with ZZTat22-57 and IL-21/IL-4/anti-CD40, indicating that this mixture stimulates B-lymphocytes able to secrete IgGs specific to fNY-ESO-1.

## Discussion

We previously showed that a specific IgM Ab response is triggered by PBMCs incubated with a fusion protein containing the transcriptional transactivator (Tat) of HIV-1 and the ZZ double Ig-binding domain derived from protein A of *Staphylococcus aureus*, suggesting that this approach might represent a new strategy of *in vitro* immunization [34]. Here, we addressed more thoroughly the potential of this new strategy. As the absence of anti-Tat IgGs indicated that ZZTat101 fails to induce the isotypic switch, we first investigated whether we could optimize our *in vitro* immunization procedure in order to trigger an IgG Ab response. To this aim, we supplemented ZZTat101 and PBMCs with a mixture containing IL-4, IL-21 and CD40L or anti-CD40 since IL-4 and IL-21 are reported to be switch factors when used in combination with either CD40L or anti-CD40 [35, 36]. We did not detect a significant amount of anti-Tat IgG in the supernatants resulting from the incubation with the CD40L/IL-4/IL-21 mixture, indicating that it does not provide an efficient switch. In contrast, we found a significant amount of IgGs with anti-CD40/IL-4/IL-21. Furthermore, Ab binding to Tat101-coated plates was inhibited by soluble Tat101, while two soluble unrelated antigens had little or no effect, indicating that this humoral immune response is specific to Tat101. Therefore, these results demonstrate that the anti-CD40/IL-4/IL-21 mixture is of value in improving our ZZTat-based *in vitro* immunization procedure and that the anti-CD40 Ab contributes more efficiently than CD40L to the isotype switch provided by IL-4 and IL-21. Moreover, and interestingly, we did not find a significant increase in the anti-Tat Ab response when we used a concentration of ZZTat101 5-fold higher than that usually used (50 and 10 μg/mL, respectively), showing that it is not necessary to increase Ag concentration in this *in vitro* immunization procedure.

To decipher the molecular mechanisms underlying the phenomenon of *in vitro* immunization, we assessed the ability of a series of protein derivatives to trigger the anti-Tat IgG Ab response when incubated with the anti-CD40/IL-4/IL-21 mixture and PBMCs. We did not detect anti-Tat IgGs with free ZZ, indicating that the double IgG-binding domain cannot initiate the specific humoral immune response. In addition, we observed that free Tat101 and the noncovalent association of Tat101 and ZZ are inefficient. Therefore, these results demonstrate that Tat101 can stimulate the *in vitro* IgG Ab response only when it is covalently coupled to ZZ. Furthermore, we did not detect anti-Tat IgGs with the ZZTat101Ø molecule, in which the basic residues of the basic-rich region of Tat have been acetylated, which indicates that this basic-rich domain is involved in the phenomenon. In contrast, the regions 1–21 and 58–101 of Tat are not crucial since the ZZTat22-57 protein, which is devoid of these two regions, triggers the humoral immune response *in vitro*. When we used a ZZTat22-57 derivative in which the seven Tat cysteines contained in the cysteine-rich region are replaced by serines (ZZTat22-57_C(22–37)S_), we did not detect anti-Tat IgGs, indicating that the cysteine-rich domain is involved in the phenomenon. In our previous work done in the absence of the anti-CD40/IL-4/IL-21 mixture, we used the same protein derivatives and reached the same conclusions for the anti-Tat IgM Ab response [34]. Therefore, our observations strongly suggest that the *in vitro* anti-Tat IgM response and IgG Ab response follow identical molecular mechanisms of activation and that the anti-CD40/IL-4/IL-21 mixture only provides the required signals to switch from IgM to IgG.

We used an ELISPOT assay to determine the frequency of B cell-producing Tat101-specific IgGs. With Tat101-coated ELISPOT plates we detected SFCs when PBMCs were incubated with the anti-CD40/IL-4/IL-21 mixture only. This result contrasts with the absence of detection of anti-Tat IgGs in the SNs resulting from the same incubation during our ELISA experiments. As the anti-CD40/IL-4/IL-21 mixture provides a strong polyclonal B-cell activation, we speculate that this discrepancy might be related to B-cell clones secreting IgGs with low affinity for the ELISPOT Tat101-coated plates. We counted significantly higher amounts of SFCs with PBMCs incubated in the presence of anti-CD40/IL-4/IL-21 and ZZTat101. Interestingly, we found a peak ELISPOT response after 11 days of incubation, which correlates with that found for the ELISA of the SNs (not shown), indicating that the level of expansion of Tat101-specific IgG+ B-cells depends on the duration of *in vitro* incubation.

To investigate whether our approach can be used to confer on an unrelated non-haptenic peptide Ag the ability to trigger a specific IgG Ab response, we prepared the ZZfNY-ESO-1Tat22-57 fusion protein containing a large fragment derived from the NY-ESO-1 cancer/testis Ag. When PBMCs were incubated with this fusion protein, we detected a significant amount of anti-fNY-ESO-1 IgG in the SN and the number of anti-fNY-ESO-1 SFCs was significantly higher than that found for PBMCs incubated with the anti-CD40/IL-4/IL-21 mixture alone.

## Conclusions

Our data demonstrate that PBMCs become able to trigger a human anti-Tat IgG Ab response when they are incubated with anti-CD40, IL4, IL21 and different ZZTat derivatives. Furthermore, they show that a NY-ESO-1 fragment raises a specific IgG Ab response when it is coupled to ZZTat. These results suggest that the ZZ-Tat based approach can be used to raise an *in vitro* humoral immune response against any Ag unrelated to Tat.

## Methods

### Antigens

#### Chemical synthesis of Tat101

Synthesis of Tat101 was performed as previously described, using the Fmoc/tert-butyl strategy on an Applied Biosystems 433A synthesizer [34]. After synthesis and completion of the Cys deprotection, the mixture was purified by HPLC on a C4 column and then characterized by mass spectrometry and amino acid analysis.

### Preparation of the fusion proteins

ZZ, ZZTat101, ZZTat22-57, ZZTat22-57_C(22–37)S_, the acetylated ZZTat101 molecule called ZZTat101Ø and ZZfNY-ESO-1Tat22-57 were produced using recombinant technology as previously described [34]. Briefly, the genetic fragments encoding the different sequences were inserted into a pCP vector using SacI/KpnI/BamHI restriction sites [40]. Then, *E. coli* BL21 (DE3pLysS) cells were transformed with the plasmids encoding ZZTat101, ZZTat22-57, ZZTat22-57_C(22–37)S,_ ZZTat101Ø or ZZfNY-ESO-1Tat22-57. Finally, the five protein constructs were expressed by IPTG induction. Bacteria were mechanically lysed in the presence of AEBSF protease inhibitor (Pefabloc, SIGMA).

The five ZZ-fusion proteins were purified by 20-min incubation at 4 °C in NaCl (2 M) and protamine sulfate (2 mg/mL) followed by a centrifugation to remove nucleic acids. Then, the samples were dialyzed in PBS-Tween 20 (0.1 %) and loaded on an affinity column grafted with Abs (IgG Sepharose 6 Fast flow #17-0969-02 Amersham).

### Purification of human peripheral blood mononuclear cells

This study was performed in accordance with the ethical standards of Declaration of Helsinki (http://www.wma.net/en/30publications/10policies/b3/index.html). Buffy coat samples from healthy blood donors, screened negative for HIV-1/2, HTLV-I/II, HCV, HBsAg, were from the Etablissement Français du Sang (EFS,Rungis, France). They were collected from anonymous donors after signing of an informed consent form and following Etablissement Français du Sang guidelines. An agreement of transfer of products stemming from blood or from its components with no therapeutic purpose is signed between the CEA and the EFS and renewed every year (number of convention: EFS: 13/EFS/101; CEA: CAJ-13-060-3). Peripheral blood mononuclear cells (PBMCs) were isolated by Ficoll Hypaque density gradient centrifugation (Histopaque 1077, Sigma-Aldrich, St. Louis, MO). Plasma from donors was isolated and stored at −20 °C for serological analysis. Cells were washed in PBS (phosphate buffer saline: 10 mM potassium phosphate pH 7.4 and 150 mM sodium chloride) supplemented with 2 mM EDTA.

### *In vitro* immunization

The *in vitro* immunization of PBMCs was performed in 96-well plates at 5 X 10^5^ cells per well in a final volume of 200 μL medium (RPMI-1640 supplemented with 2 mM L-glutamine, penicillin (50 IU/mL), streptomycin (50 μg/mL), 50 μM β-mercaptoethanol and 10 % heat-inactivated fetal bovine serum). The different cell populations were incubated *in vitro* in the presence or absence of antigens (ZZTat101, ZZfNY-ESO-1Tat22-57, ZZ or Tat101, ZZTat101 Ø, ZZTat22-57_C(22–37)S_ and ZZTat22-57) at a concentration of 10 μg/mL each and with or without an activator cocktail containing different combination of CD40L (1 μg/mL), anti-CD40 (1 μg/mL), IL4 (10 ng/mL) and IL21 (50 ng/mL). After 8, 11 or 13 days of incubation at 37 °C and 5 % CO_2_, culture supernatants were collected, and the specific IgG content was measured using an enzyme immunoassay.

### Detection of anti-Tat specific IgG using an enzyme immunoassay

96-well microtiter plates (Maxisorp, Nunc, Roskilde, Denmark) were coated with Tat101 (0.1 μg/well) in 50 mM sodium phosphate buffer (pH 7.4) at room temperature overnight. Plates were washed once with washing buffer (10 mM potassium phosphate buffer pH 7.4 containing 0.05 % Tween 20) and saturated with sodium phosphate buffer 100 mM containing 0.3 % bovine serum albumin (BSA) and 0.003 % Thymerosal and stored at 4 °C. Before use the plates were washed three times with washing buffer.

For the assessment of the presence of anti-Tat IgG, a fixed volume of supernatant was incubated in the presence or absence of a fixed amount (10 μg/mL) of different Ags (Tat101, ovalbumin, lysozyme) in microtiter plates coated with Tat101. After overnight incubation at 4 °C, plates were washed three times and a peroxidase-conjugated anti-human IgG (Jackson Immunoresearch Laboratories) was added. After a 30-min incubation, plates were washed and incubated with 2,2′-azinobis(3-ethylbenz-thiazoline-6-sulfonic acid) (ABTS). After 30 min of incubation, absorbance was read at 414 nm.

### Detection of anti-fNY-ESO-1 IgG using an enzyme immunoassay

96-well microtiter plates (Maxisorp, Nunc, Roskilde, Denmark) were coated with anti-human IgG (5 μg/well) in 50 mM sodium phosphate buffer (pH 7.4) at room temperature overnight. Plates were washed once with washing buffer (10 mM potassium phosphate buffer pH 7.4 containing 0.05 % Tween 20) and saturated with enzyme immunoassay buffer (EIA) (100 mM phosphate buffer pH 7.4 containing 150 mM NaCl, 0.1 % BSA and 0.01 % sodium azide) and stored at 4 °C. Before use the plates were washed three times with washing buffer.

For the assessment of the presence of anti-fNY-ESO-1 IgG, a fixed volume of supernatant was dispensed into the wells and reacted for two hours at room temperature. The plates were then washed before the addition of 100 μL of biotinylated f-NY-ESO-1 to each well at a concentration of 30 ng/mL. After an overnight incubation at 4 °C followed by three washing cycles, 100 μL of acetylcholinesterase (AChE; EC 3.1.1.7)-labeled streptavidin conjugate (2 Ellman units (EU)/mL [41] was added to each well. After 1 h of incubation at RT followed by three washing cycles, 200 μL of Ellman's reagent [42] was added, and the absorbance was measured at 414 nm.

### Detection of specific Ab-forming cells by ELISPOT

Nitrocellulose-backed 96-well MAHAS4510 plates (Millipore) were coated overnight at 4 °C with either Tat or NY-ESO-1 (1 μg/mL each) in 50 mM sodium bicarbonate buffer (pH 9.6). Plates were washed and blocked for 2 h at 37 °C with 10 % fetal calf serum in RPMI. PBMCs were seeded at 3 X 10^5^ cells/well and incubated for 18 h at 37 °C. Spot-forming cells (SFCs) were then detected using 1 μg/mL of biotinylated anti-human IgG (Jackson Immunoresearch Laboratories) for 1.5 h at room temperature. Ab binding was evaluated by the addition of streptavidin, alkaline phosphatase conjugate and a chromogenic alkaline phosphatase substrate (Pierce).

## Abbreviations

Tat: Transcriptional transactivator
PBMCs: Human peripheral blood mononuclear cells
Ab: Antibody
mAbs: Monoclonal antibodies
SNs: Supernatants
Ag: Antigen
OVA: Ovalbumin
AChE: Acetylcholinesterase
EU: Ellman units
SFCs: Spot-forming cells
